# Potential role of IGF-1R in the interaction between orbital fibroblasts and B lymphocytes: an implication for B lymphocyte depletion in the active inflammatory phase of thyroid-associated ophthalmopathy

**DOI:** 10.1186/s12865-024-00613-3

**Published:** 2024-05-11

**Authors:** Renyan Wang, Delu Song, Yong Zhong, Hui Li

**Affiliations:** 1 Department of Ophthalmology, Beijing Tsinghua Changgung Hospital, School of Clinical Medicine, Tsinghua University, Beijing, 102218 China; 2 Department of Ophthalmology, Shiley Eye Institute University of California, San Diego, 9415 USA; 3 Department of Ophthalmology, Peking Union Medical College Hospital, Chinese Academy of Medical Sciences & Peking Union Medical College, 1# Shuaifuyuan Wangfujing Dongcheng District, Beijing, 100730 China

**Keywords:** B lymphocytes, Insulin-like growth factor-1 receptor, Orbital fibroblasts, Thyroid-associated ophthalmopathy

## Abstract

**Background:**

Thyroid eye disease (TED) is an inflammatory process involving lymphocyte-mediated immune response and orbital tissue damage. The anti-insulin-like growth factor-1 receptor (IGF-1R) antibodies produced by B lymphocytes are involved in the activation of orbital fibroblasts and the inflammatory process of orbital tissue damage in TED. The purpose of this study was to explore the role of IGF-1R in the mechanistic connection between orbital fibroblasts and B lymphocytes in TED.

**Methods:**

Orbital fibroblasts sampled from orbital connective tissues and peripheral B lymphocytes isolated from peripheral blood, which were obtained from 15 patients with TED and 15 control patients, were co-cultured at a ratio of 1:20. The level of IGF-1R expression in orbital fibroblasts was evaluated by flow cytometry and confocal microscopy. Transient B lymphocyte depletion was induced with anti-CD20 monoclonal antibody rituximab, while the IGF-1R pathway was blocked by the IGF-1R binding protein. The expression levels of interleukin-6 (IL-6) and regulated upon activation, normal T cell expressed and secreted (RANTES) in the co-culture model were quantified via ELISA.

**Results:**

IGF-1R expression was significantly elevated in TED orbital fibroblasts compared to that of controls. A 24-h co-culture of orbital fibroblasts with peripheral B lymphocytes induced elevated expression levels of IL-6 and RANTES in each group (TED patients and controls), with the highest levels occurring in TED patients (T + T group). Rituximab and IGF-1R binding protein significantly inhibited increased levels of IL-6 and RANTES in the co-culture model of TED patients.

**Conclusions:**

IGF-1R may mediate interaction between orbital fibroblasts and peripheral B lymphocytes; thus, blocking IGF-1R may reduce the local inflammatory response in TED. Rituximab-mediated B lymphocyte depletion played a role in inhibiting inflammatory responses in this in vitro co-culture model, providing a theoretical basis for the clinical application of anti-CD20 monoclonal antibodies in TED.

## Background

Thyroid eye disease (TED), the most common extrathyroidal condition of Grave’s disease (GD), is an inflammatory process involving lymphocyte-mediated immune responses and orbital tissue damage [1]. In the immune response, activated T lymphocytes secrete large amounts of inflammatory cytokines, such as interleukin (IL)-6, 8, and 16, etc., to mediate the process of tissue damage and destruction, which subsequently stimulates B lymphocytes to produce autoantibodies. Anti-insulin-like growth factor-1 receptor (IGF-1R) antibodies produced by B lymphocytes have been shown to be involved in the activation of orbital fibroblasts in patients with TED [2, 3]. Hence, inhibition of IGF-1R has become a therapeutic target for TED [4].

The role of B lymphocytes during acute attacks of TED has previously been demonstrated. B lymphocytes may play a more important role than simply serving as a source of antibodies in the development of TED, as they may also contribute to initiating the process of autoimmune reactions in an earlier phase of TED development [5]. Orbital fibroblasts are key effector cells in the development of TED, which can cause hypertrophy of extraocular muscles and hyperplasia of adipose tissue. Under the stimulation of autoantibody IgG secreted by B lymphocytes, orbital fibroblasts in TED can secrete more hyaluronic acid, which leads to a sharp elevation in the volume of orbital contents and orbital pressure [6]. Moreover, when stimulated by autoantibody IgG, fibroblasts isolated from the skin of GD patients can produce more cell chemoattractants and regulated upon activation, normal T cell expressed and secreted factors (RANTES) through IGF-1R mediation [7]. However, few reports have explored the interaction between orbital fibroblasts and B lymphocytes in patients with TED. Therefore, we established a new in vitro model that involves the co-culturing of orbital fibroblasts with peripheral blood B lymphocytes, which in this study were sampled from TED patients and control patients, to simulate the growth environment of orbital fibroblasts in the TED orbit and explore the role of B lymphocytes in the active inflammatory phase of TED.

## Methods

### Patient selection and evaluation

Fifteen patients with active TED (clinical activity score (CAS) ≥ 3) [8], and 15 negative controls (healthy subjects without any known ophthalmopathy before ocular trauma) were enrolled in this study, which was conducted in the Department of Ophthalmology at Peking Union Medical College Hospital in Beijing, China. TED was diagnosed according to Bartley’s diagnostic criteria [9]. A thorough ophthalmic examination was performed, including vision acuity, slit-lamp, ophthalmoscopy, intraocular pressure, and Hertel exophthalmometry. Eyelid retraction, soft tissue involvement, proptosis, diplopia, extraocular muscle motility, lagophthalmos, optic nerve damage, orbital pain, eye irritation symptoms, and photophobia were assessed. A laboratory test of the thyroid function and autoimmunity was conducted for each patient. The CAS (range 0–7) was assessed, and the classification and severity of TED was graded using the NOSPECS system [8] and EUGOGO guidelines [10].

The inclusion criteria for TED patients were (1) the diagnosis of TED met Bartley’s diagnostic criteria; (2) patients who were 20–65 years old; (3) patients who had no other eye diseases or history of eye surgery; (4) patients had no hypertension, diabetes, hyperlipidemia, or other systemic diseases; (5) myopia degree < − 6.00D; and (6) patients had no contraindications for the use of glucocorticoids and orbital decompression surgery. The exclusion criteria for TED patients were (1) patients who were > 65 years old or < 20 years old; (2) patients who had a history of orbital decompression or strabismus correction surgery; (3) patients who did not cooperate with eye examinations, and (4) patients with cloudy refractive media that was difficult to be imaged. The inclusion criteria for the control subjects were (1) patients who were 20–65 years old; and (2) patients who underwent surgery for primary concomitant strabismus or traumatic eye-ball rupture. The exclusion criteria were (1) patients who were 20–65 years old; (2) patients who could not or refused to cooperate with the examination; (3) patients with a history of uveitis or fundus diseases; and (4) patients with a history of autoimmune disease.

The treatment strategy for patients with lagophthalmos and corneal damage, and those with optic nerve damage due to high orbital pressure or hypertrophy of the ocular muscles was as follows. Intravenous infusion of methylprednisolone 0.5 g/day for 3 days, followed by oral administration of steroids. During the process of steroids dose reduction, the visual function was checked. If visual acuity deteriorated or did not improve, orbital decompression surgery would be performed two weeks later. All of the patients underwent balanced decompression of the inner and outer walls of the orbit under nasal endoscopy, followed by postoperative hemostasis and anti-inflammatory treatment. Steroid treatment continued to one month after surgery. A written informed consent was obtained from each subject. This study was approved by the Institutional Review Board Committee of Peking Union Medical College Hospital.

### Tissue procurement and cell culture

Orbital connective tissue samples were obtained from surgical waste during orbital decompression surgery performed for therapeutic purposes in 15 patients in the active status of TED, or during strabismus correction or enucleation surgery performed for ocular trauma in 15 patients without TED used as controls in this study. Intraoperative resection of orbital connective tissue sampled from these 30 patients was performed to generate independent primary cultures of orbital fibroblasts. Samples were not pooled at any time during these experiments. The obtained orbital connective tissue samples were removed from adipose tissue and large blood vessels under the microscope. After rinsing with PBS solution containing penicillin and Streptomycin, they were cut into tissue blocks of about 1mm^3^ with scissors. The cut tissue blocks were transferred to a 5 cm Petri dish with micro tweezers, and placed evenly. After the tissue blocks were slightly dried, they were incubated at 37 °C under a humidified 5% CO_2_ atmosphere in a PRMI-1640 culture medium containing 10% fetal bovine serum. Primary cultures were used between passages 3 to 7 for each experiment. Peripheral blood samples were collected from patients with TED and controls during a routine examination upon admission. B lymphocytes obtained from subjects’ blood samples were enriched and purified using an immunomagnetic beads separation technique (Miltenyi Biotec, USA), and then analyzed by flow cytometry. The suspension of B lymphocytes isolated from peripheral blood was transferred to two Eppendorf tubes, each containing approximately 2 × 10^5^ B lymphocytes. The two tubes were centrifuged at 3,000–4,000 rpm for 5 min; then, the supernatant was discarded and the tubes were sealed with 1% BSA. Next, 5 µL anti-human PE labeled CD19 antibody was added into one tube under dark conditions and incubated at 4 °C for 30 min. The other tube was a negative control. Afterwards, the cells were rinsed twice with 200 µL stain buffer, and finally fixed with 1% paraformaldehyde for testing.

### Co-culture of orbital fibroblasts with B lymphocytes

Orbital fibroblasts used between passages 3 to 7 were digested using trypsin and then cultured by direct adherent culture methods one day prior to co-culturing with peripheral blood B lymphocytes in a ratio of 1:20. On day 1 of co-culturing, 1 µg/mL CpG oligodeoxynucleotides (CpG 2006, Sangon Biotech, Shanghai) was added to stimulate B lymphocyte activity. After 72 h of co-culturing, cells were harvested and divided into four groups: T + T group (both samples obtained from TED patients), T + N group (B lymphocytes sampled from TED patients and orbital fibroblasts sampled from control patients), N + T group (B lymphocytes sampled from control patients and orbital fibroblasts sampled from TED patients), and N + N group (both samples obtained from control patients).

### IGF-1R detection

The levels of IGF-1R expression in orbital fibroblasts obtained from TED patients in comparison with those of controls were analyzed quantitatively by using flow cytometry and an immunofluorescence staining technique. Orbital fibroblasts were prepared as a single-cell suspension with an approximate cell density of 5 × 10^5^. After centrifugation, cells were added to 2 µL FITC-labeled human anti-IGF-1R antibody (Santa Cruz, USA) and incubated at 4 °C for 30 min. Flow cytometry detection was performed after fixation using 1% paraformaldehyde. For immunofluorescence, cells were grown on polylysine-coated glass chamber slides and fixed with 2% paraformaldehyde for 30 min at room temperature. After treatment with 0.2% Triton X-100 at room temperature for 30 min, a monoclonal anti-IGF-1R antibody (5 µg/mL, 5 µL) was incubated overnight at 4 °C. Next, an FITC-labeled goat anti-mouse IgG was added. The fluorescence was visualized using a confocal microscope (40×) and then stained with 4’,6-diamidino-2-phenylindole for 5 min.

### Assessment of interleukin-6 and RANTES levels

The B-lymphocyte depletion effect was mediated by anti-CD20 monoclonal antibody rituximab (RTX, Roche, Shanghai, China). IGF-1R binding protein was used to block the IGF-1R pathway. CpG oligodeoxynucleotides were used to stimulate B lymphocyte activity. An MTS assay (Promega, USA) was used to detect cell proliferation activity in cells treated with different concentrations of RTX (1 mg/mL, 0.8 mg/mL, 0.6 mg/mL, 0.4 mg/mL or 0.2 mg/mL), the IGF-1 binding protein, or CpG. On day 1 of co-culturing orbital fibroblasts with B lymphocytes, 1 mg/mL RTX, 5 µL/mL IGF-1 binding protein, and 1 µg/mL CpG were added to the medium, according to the results of the MTS assay (data not shown). A negative control group was created for each drug treatment. The supernatant of co-cultured cells was collected after 48 h and centrifuged at 1,000 g for 10 min to remove debris. The expression levels of interleukin-6 (IL-6, RayBiotech, USA) and RANTES (RayBiotech, USA) were quantified in triplicate via an enzyme-linked immunosorbent assay (ELISA) according to the manufacturer’s protocols.

### Statistical analysis

Continuous data are presented as the mean and standard deviation, whereas categorical data are presented as percentages. Continuous variables were analyzed using a *t*-test. Categorical data among groups were analyzed using a Chi-square test. The sum of the ranks of data among groups was calculated using a Wilcoxon rank sum test. We used R software (version 4.0.4) to analyze the data. *P* values less than 0.05 were considered to be statistically significant.

## Results

### Clinical and biomedical characteristics of the study subjects

A total of 15 patients with TED and 15 age- and gender-matched control patients met the inclusion criteria for this study. The average clinical activity score for the 15 TED patients was 4.83 ± 1.7 (range 3–7). General and biomedical characteristics of the study subjects are summarized in Table 1. Clinical characteristics of the TED patients are summarized in Table 2. The TED patients and control patients did not differ with respect to age, gender, body mass index, total triiodothyronine, total thyroxine, thyroid-stimulating hormone (TSH), or smoking status (*p* > 0.05), while significant differences were detected in thyrotropin receptor antibody, TSH-receptor antibody, free triiodothyronine, and free thyroxine levels between the TED patients and the control subjects (*p* < 0.01).

**Table 1:** General and biochemical characteristics of the study subjects

General information	TED patients (*n* = 15)	Controls (*n* = 15)	P value
Age—yr	47.9 ± 10.3	46.5 ± 9.8	0.083
Gender—no. (%) Male Female	7 (46.7%) 8 (53.3%)	6 (40.0%) 9 (60.0%)	0.713
Body mass index (kg/m^2^)	25.3 ± 3.2	26.4 ± 3.8	0.091
Total triiodothyronine (µM/l)	1.9 ± 0.2	2.0 ± 0.4	0.098
Total thyroxine (µM/l)	96.9 ± 11.9	93.8 ± 14.3	0.074
Thyroid-stimulating hormone (mU/l)	1.8 ± 0.4	1.5 ± 0.2	0.078
Smoking status Currently smoke	4 (26.7%)	5 (33.3%)	0.628
Previously smoked Never smoked	7 (46.7%) 4 (26.7%)	3 (20.0%) 7 (46.7%)	
TSH-receptor-antibody (mU/L)	43 ± 3.8	1.5 ± 0.3	0.001
Free triiodothyronine (pmol/L)	15 ± 1.9	3.4 ± 0.5	0.008
Free thyroxine (pmol/L)	27.8 ± 0.4	14.7 ± 2.3	0.006
Type of autoimmune thyroid disorder			
Hyperthyroidism	10/15		
Hypothyroidism	2/15		
Hashimoto’s thyroiditis	2/15		
Thyroid cancer	1/15		

**Table 2:** Ophthalmology examination/evaluation of TED in the 15 patients

Examination/evaluation	Signs/grade	Eyes
Proptosis	Yes	15
Diplopia	Yes	12
Eyelids	Edema	11
Visual acuity	Decreased	15
Conjunctiva	Congestion	15
	Edema	12
Cornea	Punctate infiltration	4
	Ulcer	8
Elevated intraocular pressure	Mild	4
	Moderate	6
	Severe	5
Ptosis	Yes	12
Upper eyelid retraction (mm)	< 2	1
	2 ~ 4	2
	> 4	12
Lower eyelid retraction (mm)	< 2	4
	2 ~ 4	2
Extraocular muscle motility	Partly limited	8
	Fixed	4
CAS	3–7 (4.83 ± 1.7)	15
NOSPECS	5	7
	6	8
EUGOGO	Very severe	15

CAS, clinical activity score; NOSPECS, no signs or symptoms, only signs, soft tissue involvement, proptosis, extraocular muscle involvement, corneal involvement, sight loss; EUGOGO, European group on Graves’ orbitopathy

### Establishment of the co-culture model using orbital fibroblasts and B lymphocytes

**Fig. 1:**
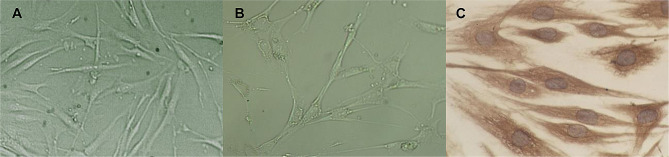
Cultures of primary orbital fibroblasts sampled from patients with thyroid-associated ophthalmopathy (TED) and controls. (**A**) Phase-contrast light micrographs of cultured orbital fibroblasts sampled from TED patients. (**B**) Phase-contrast light micrographs of cultured orbital fibroblasts sampled from control patients. (**C**) Immunopositivity for vimentin in primary orbital fibroblast cultures

**Fig. 2:**
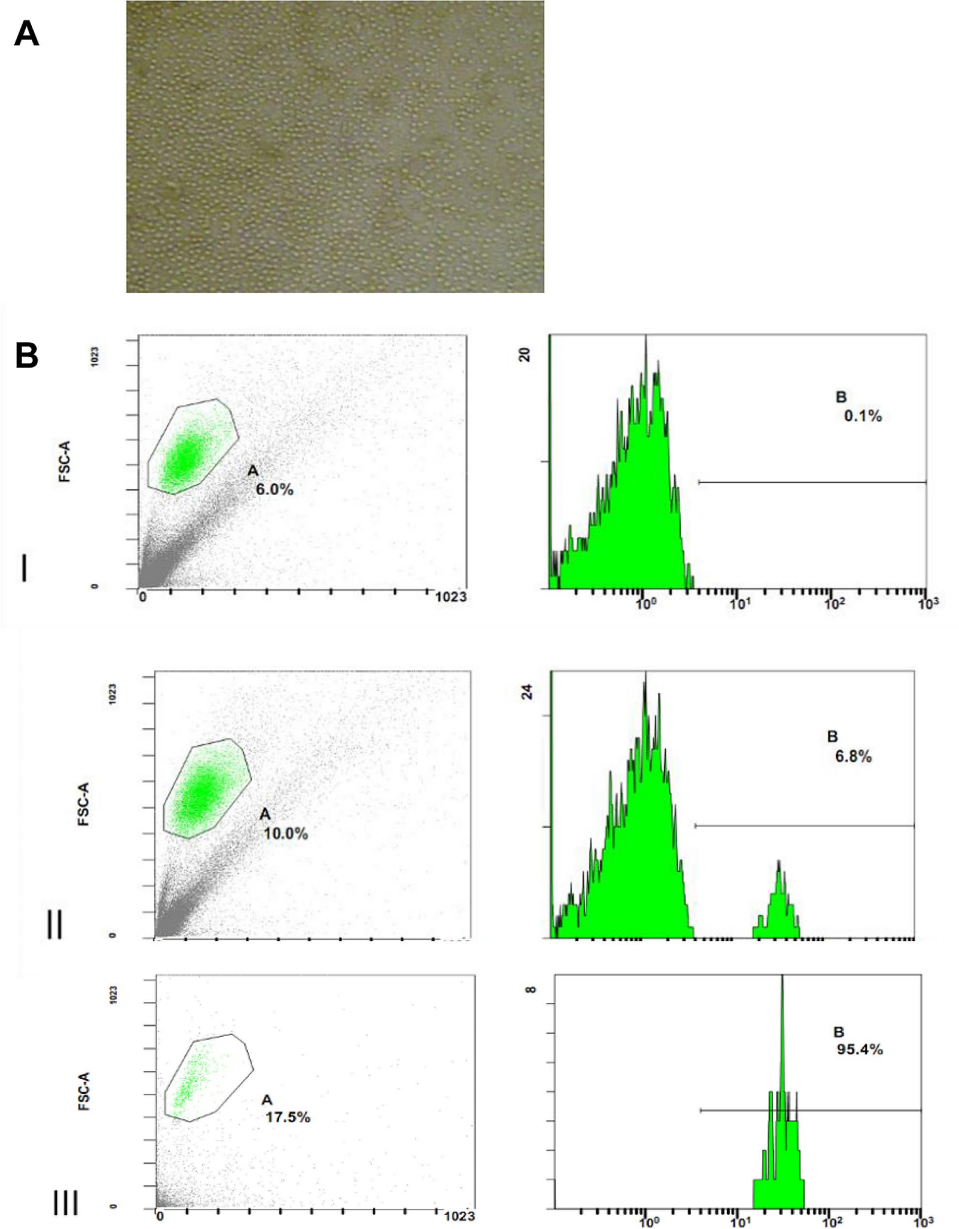
The images show the purified peripheral blood B lymphocytes (**A**) under a phase-contrast light microscope and their characterization with flow cytometry (**B**). The purity of B lymphocytes was detected using flow cytometry. B(I), negative control; B(II), content of B lymphocytes in peripheral blood before sorting; B(III), purity of sorted B lymphocytes

**Fig. 3:**
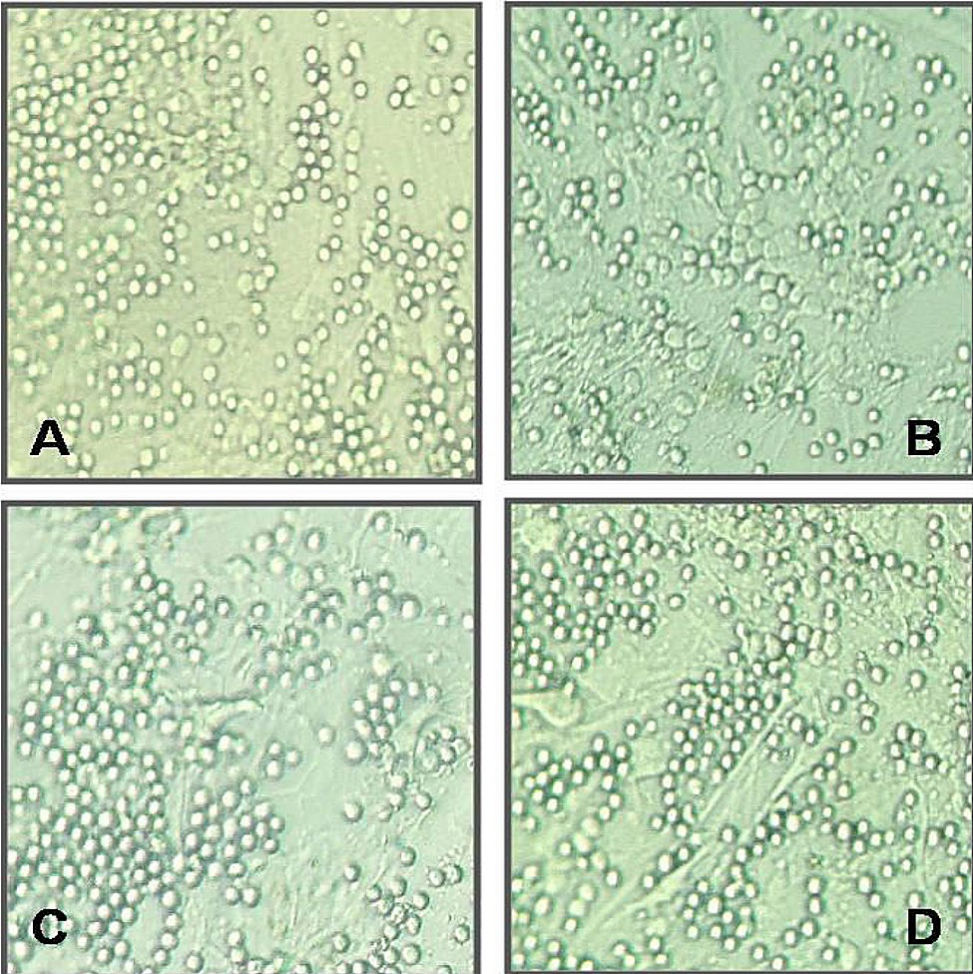
Morphologies of orbital fibroblasts and B lymphocytes co-cultured for 72 h under a phase-contrast light microscope. (**A**) Co-culture model of TED patients (T + T group, both samples obtained from TED patients). (**B**) Co-culture model of B lymphocytes in TED patients and orbital fibroblasts in controls (T + N group, B lymphocytes sampled from TED patients and orbital fibroblasts sampled from control patients). (**C**) Co-culture model of B lymphocytes in controls and orbital fibroblasts in TED patients (N + T group, B lymphocytes sampled from control patients and orbital fibroblasts sampled from TED patients). (**D**) Co-culture model of B lymphocytes and orbital fibroblasts in controls (N + N group, both samples obtained from control patients)

In Fig. 1, we confirmed that the primary cultures obtained from TED patients and controls were orbital fibroblasts according to morphology and immunopositivity for vimentin, which shows the characteristics of fibroblasts. The cultures were immunonegative for desmin, myoglobin, cytokeratin, and S-100, and excluded the sources of smooth muscle, myocardium, striated muscle, neurocytes, and skin melanocytes. The purified peripheral blood B lymphocytes and their characterization with flow cytometry are shown in Fig. 2. The amount of peripheral blood B lymphocytes in the negative control was 0.1%. The amounts of B lymphocytes before and after immunomagnetic beads separation were 6.8% and 95.4%, respectively. After 72 h of co-cultivation, there were no significant changes in the morphologies of the orbital fibroblasts and B lymphocytes, and there were no significant differences in cell morphology among the four groups under a microscope (Fig. 3).

### Higher IGF-1R expression in TED orbital fibroblasts than in controls

**Fig. 4:**
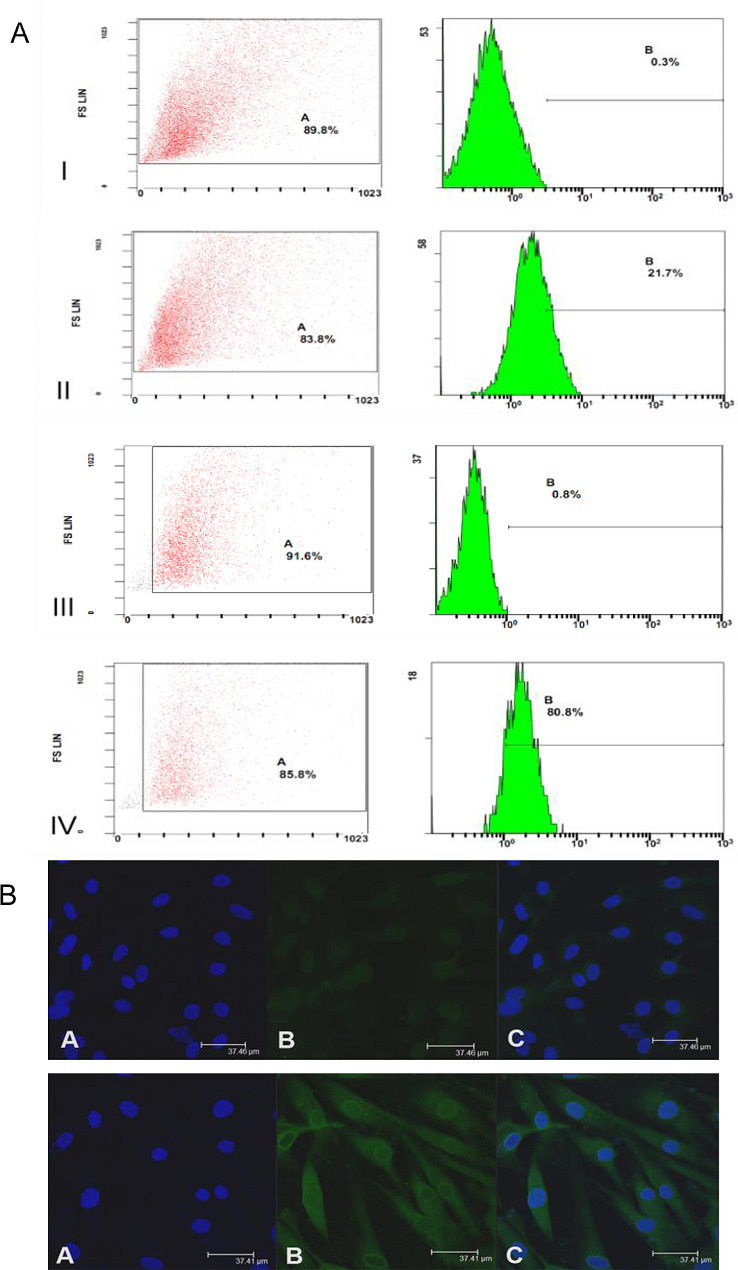
Insulin-like growth factor-1 receptor (IGF-1R) expression of orbital fibroblasts in TED patients and controls. (**A**) Flow cytometric analysis demonstrated that the IGF-1R content of orbital fibroblasts was 21.7% in control patients (II) and 80.8% in TED patients (IV), respectively, while (I) and (III) were negative controls. (**B**) A representative confocal picture showing elevated IGF-1R expression in the orbital fibroblasts from TED patients compared to that of controls

IGF-1R levels in the orbital fibroblasts obtained from TED patients and controls were detected using flow cytometry and a confocal laser scanning microscope. As shown in Fig. 4, TED orbital fibroblasts had higher IGF-1R levels than those of the controlled orbital fibroblasts.

### Co-cultures of samples from TED patients induced the highest levels of IL-6 and RANTES

**Fig. 5:**
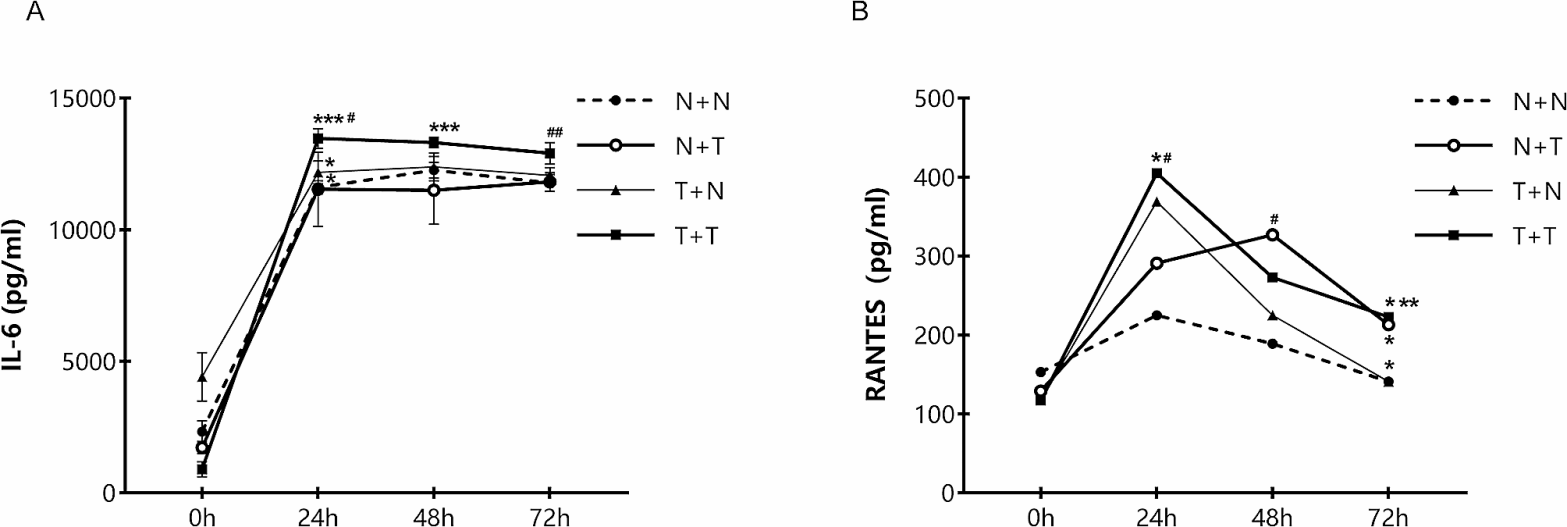
Interleukin-6 (IL-6) and regulated upon activation normal T cell expressed and secreted factors (RANTES) expression levels after 72 h of co-culturing four groups. (**A**) Rapid increase of IL-6 levels in four groups within 24 h, with significantly higher values for the T + T group at each detection time point than those of the other three groups. (**B**) Increased expression of RANTES within 24 h of co-cultivation in all groups, with the highest concentration of RANTES occurring in the T + T group 24 h after co-culture. Data are represented as the mean and standard deviation of three independent experiments. *P < 0.05, **P < 0.01, ***P < 0.001

As shown in Fig. 5A, the levels of IL-6 in the four groups rapidly increased within 24 h of co-cultivation, and then the upward trend significantly slowed down after 24 h. The values of the T + T group at each detection time point were significantly higher than those of the other three groups at the same time points, and the highest level of IL-6 in the T + T group was observed 24 h after co-culturing. Figure 5B shows an increased expression of RANTES within 24 h of co-cultivation in all groups, but the expression showed a decreasing trend 24 h after co-cultivation. Similar to IL-6 expression, the T + T group had the highest concentration of RANTES at 24 h after co-culturing.

### Inhibition of IGF-1R and B lymphocytes reversed the increased trends of IL-6 and RANTES in TED orbital fibroblasts

**Fig. 6:**
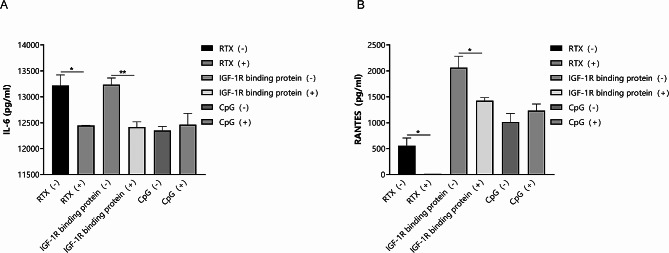
Effects of rituximab (RTX), IGF-1R binding protein, and CpG oligodeoxynucleotides on IL-6 and RANTES expression in the co-culture of orbital fibroblasts with B lymphocytes in TED patients (T + T group). (**A**) IL-6 levels in the T + T group reduced after 48 h of cultivation with RTX and the IGF-1R binding protein. (**B**) RANTES levels in the T + T group reduced after 48 h of cultivation with RTX and the IGF-1R binding protein. Data are represented as the mean and standard deviation of three independent experiments. *P < 0.05

The results of the MTS assay showed that the inhibitory effect of RTX on B lymphocytes was concentration- and time-dependent within a certain range. Based on considerations of cytotoxicity and inhibitory effects, the optimal concentration of RTX was established as 1 mg/mL RTX on the same day that co-culturing of orbital fibroblasts with B lymphocytes in TED patients (T + T group) was performed. Similarly, 5 µL/mL IGF-1R binding protein and 1 µg/ml CpG were added to the medium, respectively, according to the results of the MTS assay (data not shown). The ELISA results show that 48 h of cultivation, the levels of IL-6 and RANTES were both significantly reduced in groups that received treatment with RTX or the IGF-1R binding protein, and the addition of CpG had no influence on the concentrations of IL-6 and RANTES (Fig. 6).

## Discussion

In the present study, we established an in vitro experimental model of TED by co-culturing orbital fibroblasts with peripheral B lymphocytes. We found that the expression of IGF-1R in orbital fibroblasts in patients with TED was significantly higher than that of the controls. In addition, IL-6 and RANTES expression levels were highest in the co-culture model of the T + T group (both of the cells used in the co-culture were obtained from TED patients). RTX and IGF-1R binding protein each significantly reversed the increased trend of IL-6 and RANTES in the T + T group. Our findings indicate that IGF-1R may be involved in the mechanistic link between orbital fibroblasts and B lymphocytes in the acute phase of inflammatory response in TED. B lymphocyte-depleting agent RTX showed a good anti-inflammatory effect in this in vitro co-culture model, which further supports the clinical application of the anti-CD20 monoclonal antibody in TED.

The characteristic pathological manifestations of TED include orbital fibroblast proliferation, adipose tissue hyperplasia, extraocular muscle hypertrophy, and, ultimately, fibrosis, accompanied by inflammatory cell infiltration [11]. Because the orbital volume in animals is not limited by the orbital wall, it is not possible to examine orbital signs in vivo, although there is a lack of effective methods that can maintain the serum levels of thyroxine in animal models that would allow for visual observations of the characteristic changes. Therefore, the commonly used research method for TED is in vitro cultivation of orbital fibroblasts. However, it is impossible to study the interaction between cells solely by culturing orbital fibroblasts. Therefore, this study established a new co-culture model that evaluates the interaction of orbital fibroblasts with B lymphocytes isolated from the peripheral blood of TED patients in comparison to those obtained from controls. This model has certain pertinence for autoimmune diseases, as it can better simulate the growth microenvironment of orbital fibroblasts in the presence of B lymphocytes in TED patients, thereby providing direct evidence for investigating the interactions between orbital fibroblasts and B lymphocytes in the pathogenesis of TED.

CpG can activate a variety of immune effector cells and plays a strong role in activating B lymphocytes without changing biological characteristics [12]. Therefore, when establishing this co-culture model of TED, we added a certain concentration of CpG to activate B lymphocytes and slow down the apoptosis of B lymphocytes in vitro.

IGF-1R is involved in the regulatory activities of various cell proliferation and metabolism processes [13–16]. Han R et al. reported that IgG in serum stimulated the synthesis of hyaluronic acid in fibroblasts from patients with GD, and fibroblasts obtained from GD patients’ skin had higher levels of IGF-1R than those obtained from the skin of healthy individuals. Therefore, they speculate that antibodies targeting IGF-1 receptors in the serum of TED patients act on orbital fibroblasts, causing them to either secrete more hyaluronic acid or differentiate into adipocytes [17]. Raymond et al. found that the IGF-1R receptor was not only highly expressed in the skin fibroblasts and T lymphocytes of GD patients but was also abnormally elevated in B lymphocytes [18]. IGF-1R has been recognized as an important autoantigen in the development of TED [7], and teprotumumab, an IGF1-R antagonist, is considered to be a milestone in the future management of active GD [19, 20]. Our finding in this study proved that the orbital fibroblasts of TED patients also expressed higher levels of IGF-1R than those of normal individuals, which is similar to the finding of higher levels of IGF-1R on the fibroblasts obtained from GD patients’ skin reported by a previous study [17]. Gianoukakis AG et al. found that IgG in GD patients induced the expression of IL-16 and RANTES in skin fibroblasts, and these cytokines induced chemotaxis and activated T lymphocytes in GD patients, allowing for sustained autoimmune responses. However, IGF-1 and its receptor-specific ligand-binding protein inhibited the induction of these cytokines [7]. In our co-culture model, we obtained similar results, indicating that B lymphocytes act on orbital fibroblasts through IGF-1R, which mediates the development of TED, and that blocking IGF-1R can reduce the local inflammatory response of TED.

We also found that the levels of IL-6 and RANTES in the T + T group were higher than those in the other three groups at 24 h, 48 h, and 72 h after co-cultivation, and the level was highest at 24 h. The increase in IL-6 and RANTES indicated a significant local inflammatory response. During the early stage of immune response of TED, local inflammatory reactions involved chemotactic immune cells rapidly migrating to the orbital tissues, thereby activating the body’s immune cells. These findings are consistent with the clinical course characteristics of the samples we collected. TED patients with recurrent local inflammation and a clinical activity score ≥ 3 were included in this study. Therefore, the abnormal increase in IL-6 and RANTES suggests a correlation between the activity and severity of the disease.

RTX is a B lymphocyte inhibitor of chimeric mouse/human monoclonal antibodies that specifically bind to CD20 antigens on the B lymphocytes membrane. CD20 molecular blockers can block the proliferation of B lymphocytes and reduce the number of pre-B lymphocytes and mature B lymphocytes [21]. Salvi et al. reported that B lymphocyte production and the clinical activity score decreased in a TED patient, although the patient’s thyroid function did not improve [22]. Some clinical studies on RTX treatment of TED have shown that RTX has no effect on thyroid function, despite causing a significant improvement in the clinical activity score and eye performance [23–25]. However, the reliability of clinical trial results related to RTX treatment of TED is influenced by their small sample sizes, varying degrees of patient conditions, and different natural outcomes of individual disease courses [26]. Our findings indicated that by inhibiting B lymphocytes in TED, RTX can reduce the stimulation of orbital fibroblasts and the generation of IL-6 and RANTES, inhibit inflammatory reactions in TED, block T lymphocytes and other immune cells from being chemotactic to the lesion, and interfere with the progression of pathological processes. The application of the B lymphocytes inhibitory drug RTX in TED may have a certain effect on inhibiting local inflammatory reactions in the disease. The limitation of this study was the small sample size, so further studies are warranted to reveal the cell interaction in TED patients of various stages and circumstances.

## Conclusions

In summary, this study established an in vitro experimental model of TED by co-culturing orbital fibroblasts with peripheral B lymphocytes, which lays the foundation for future investigations of the pathogenesis of TED based on the interaction between orbital fibroblasts and B lymphocytes. One finding of this study is that blocking IGF-1R reduced the local inflammatory response in this co-culture model, which indicates that IGF-1R may be involved in the interaction between orbital fibroblasts and B lymphocytes. The other main finding is that RTX inhibited inflammatory reactions in the co-culture model of TED, providing a theoretical basis for the clinical application of anti-CD20 monoclonal antibody-mediated B lymphocyte depletion as a treatment for TED.

## Data Availability

The datasets used and/or analyzed during the current study are available from the corresponding author on reasonable request.

## Abbreviations

TED: Thyroid eye disease
IL-6: interleukin-6
RANTES: regulated upon activation, normal T cell expressed and secreted
GD: Grave’s disease
IGF-1R: insulin-like growth factor-1 receptor
ELISA: enzyme-linked immunosorbent assay
